# Hawksbill Sea Turtle (*Eretmochelys imbricata*) Blood and Eggs Organochlorine Pesticides Concentrations and Embryonic Development in a Nesting Area (Yucatan Peninsula, Mexico)

**DOI:** 10.3390/toxics11010050

**Published:** 2023-01-03

**Authors:** Patricia I. Salvarani, Luis R. Vieira, Jaime Rendón-von Osten, Fernando Morgado

**Affiliations:** 1 Department of Biology and the Centre for Environmental and Marine Studies (CESAM), University of Aveiro, 3810-193 Aveiro, Portugal; 2 Interdisciplinary Centre of Marine and Environmental Research (CIIMAR), University of Porto, Terminal de Cruzeiros do Porto de Leixões, Av. General Norton de Matos s/n, 2250-208 Matosinhos, Portugal; 3 School of Medicine and Biomedical Sciences (ICBAS), University of Porto, Rua de Jorge Viterbo Ferreira, 228, 4050-313 Porto, Portugal; 4 Instituto Epomex, Universidad Autónoma de Campeche, Av Augustin de Melgar y Juan de la Barrera s/n, Campeche 24039, Mexico

**Keywords:** organochlorine pesticides, sea turtles, nesting populations, hawksbill turtles (*Eretmochelys imbricata*), maternal transfer, Yucatan Peninsula, Mexico

## Abstract

Environmental contaminants with chemical origins, such as organochlorine pesticides (OCPs) have major impacts on the health of marine animals, including sea turtles, due to the bioaccumulation of those substances by transference throughout the food chain. The effects of environmental pollution on the health of marine turtles are very important for management strategies and conservation. During recent decades, the south Gulf of Mexico and the Yucatan Peninsula have suffered from increasingly frequent disturbances from continental landmasses, river systems, urban wastewater runoff, port areas, tourism, industrial activities, pesticides from agricultural use, and other pollutants, such as metals, persistent organic pollutants (POPs) and hydrocarbons (from the oil industry activities), which contaminate water and sediments and worsen the environmental quality of the marine ecosystem in this region. In this study, we assessed the concentrations of OCPs in the blood and eggs of 60 hawksbill turtles (*Eretmochelys imbricata*) nesting at the Punta Xen turtle camp, and their effects on the nesting population’s reproductive performance: specifically, maternal transfer and embryonic development were analyzed. Hematologic characteristics, including packed cell volume, white blood cell count, red blood cell count, and haemoglobin levels, and plasma chemistry values, including creatinine, blood urea nitrogen, uric acid, triglyceride, total cholesterol and glucose, were also measured. The general health of the turtles in this study, as well as their levels of urea, serum creatinine, glucose, uric, acid, cholesterol, and triglyceride, fell within normal ranges and was similar to other normal values, which could indicate the turtles’ good energy levels and body conditions for nest-building activity, with all of the turtles able to successfully come ashore to nest. All the same, the obtained results also indicate that OCPs affect the nesting and reproductive performance of the hawksbill turtles, as well as their fertility and the development of the population of eggs and reproductive performance, specifically in terms of maternal transference and embryonic development. There were significant differences in the concentrations of OCPs (ΣHCHs and ΣDienes) between maternal blood and eggs, indicating that these chemicals are transferred from nesting females to eggs and, ultimately, to hatchlings. OCPs may, therefore, have an effect on the health and reproductive performance of hawksbill turtles, both in terms of their fertility and egg development. Conservation strategies need to be species-specific, due to differences in feeding, and address the reasons for any decline, focusing on regional assessments. Thus, accurate and comparable monitoring data are necessary, which requires the standardization of monitoring protocols.

## 1. Introduction

Sea turtles are among the oldest animals on Earth, their origins dating back more than 150 million years [1,2]; additionally, they are some of the most widely distributed vertebrates on the planet [3,4]. The adults of some species can be found throughout tropical, temperate, and subarctic waters, and regularly migrate hundreds or thousands of kilometers between foraging areas and nesting grounds [3,5]. They have a long-life expectancy, late maturity, slow reproductive rates, vast geographic ranges, and spend all their lives at sea, coming to beaches exclusively to lay eggs [6,7,8,9]. Their movements during spawning and feeding between different habitats (seagrass beds, coral reefs, ocean waters, and sandy beaches) are considered especially important for energy transfer and nutrient recycling in aquatic systems [4,10,11]. Marine turtles have a particularly important ecological role in the coastal ecosystem, both as consumers (seaweed, seagrass, sponges, tunicates, crustaceans, cnidarians) and as prey (eggs, juveniles, and adults) [11,12], thus occupying different concentrations in the food chain. Marine turtles are also reptiles with slow growth rates and long-life cycles, and are often used as models for evolutionary studies of adaptation to different environmental conditions, since they are extremely susceptible to several anthropogenic activities at all phases of their life cycle [13,14,15,16]. During their life cycle, sea turtles face various challenges in the fight for survival and are subject to many biotic, abiotic, and anthropogenic threats [17,18,19,20]. Many marine turtle populations are declining worldwide at alarming rates [8,20] and are considered globally threatened or endangered [20], and are nearing extinction [20,21]. Global sea turtle conservation programs have been developed and are currently ongoing; these programs have environmental, biological, and socio-economic dimensions. Various institutions and government bodies, spanning multiple geopolitical boundaries, and agreements at local, national, and international scales, have been established to ensure the assessment of risks and threats and the development of conservation strategies, to define conservation and management priorities, and to develop ecological information to assist in decision making [21,22,23,24,25,26]. 

The Gulf of Mexico and the Yucatan Peninsula represent an area of vital importance for the mating, breeding, foraging, and developmental habitats of six of the seven existing sea turtle species in the world [27,28,29,30]: the green turtle (*Chelonia mydas*), the loggerhead turtle (*Caretta caretta*), the olive ridley turtle (*Lepidochelys olivacea*), Kemp’s ridley turtle (*Lepidochelys kempii*), the hawksbill turtle (*Eretmochelys imbricata*), and the leatherback turtle (*Dermochelys coriacea*) [31,32,33]. This area is highly vulnerable to environmental and anthropogenic pressures [34,35,36] that cause the degradation of and changes to the quality of water and sediments, and produce pollution and contaminants, which may significantly affect turtles’ health and development [33,37,38]. During the last few decades, due to rapid increases in population, urbanization, agriculture, industrialization, fisheries, and leisure activities [39,40,41,42], this region has suffered from various disturbances. Moreover, significant contamination is visible, such as that produced by pesticides, trace metals, phosphorous, PCBs, and polycyclic aromatic hydrocarbons that can be absorbed and concentrated by sediments, water, and suspended matter in this aquatic system [42,43,44]. Anthropogenic pollution, oil spills, and chemical runoff seriously contribute to the degradation of water and sediment quality in this region, and may have severe impacts on sea turtles [32,33,36,38,45,46,47]. Recently, several works have reported the effects of environmental [30,48,49,50] and anthropogenic disturbances [51,52,53,54,55] on turtle populations dynamics and distribution, migration corridors, species co-occurrence, the oxidative stress of nesting females, genetic structures, and connectivity between nesting and foraging areas and global threats for foraging habitats, namely threats for foraging habitats in the Gulf of Mexico. Chemical contamination is one of the biggest threats in the region for the turtles’ reproduction and nesting migratory movements, and is responsible for the degradation of foraging habitats and the occurrence of embryonic deformities [45,48,56,57]. Levels of contaminant exposure in marine turtles may vary according to the level of contamination and the time spent on foraging grounds [58], although the exact impact of chemical pollutants on sea turtles’ health is unknown and there is no information available on their toxicological effects or thresholds for any marine reptiles. Due to the biogeographic relevance of this region for the turtles’ breeding, mating, foraging, and developmental habitats, several sea turtle conservation programs and turtle monitoring and conservation marine identification programs for marine key areas have been developed across the Caribbean and Yucatán Peninsula to ensure the conservation of these critically endangered species [59,60,61,62,63,64]. 

Environmental contaminants, such as organochlorine contaminants (OCs) including organochlorine pesticides (OCPs) and polychlorinated biphenyls (PCBs), are highly persistent lipophilic organic pollutants that were first introduced into the environment in the late 1940s [65]. There is considerable evidence that, due to their high persistence in the environment, and their hydrophobicity and resistance to environmental degradation, OCPs can be absorbed and concentrated by sediments, water and suspended matter, biomagnified in the food webs, and bioaccumulated in organisms, including marine reptiles and mammals through direct and trophic exposure [66,67,68,69]. Several studies emphasize the relationship that arises from the sea turtles’ long-life cycles and their high capacity for OCP bioaccumulation [58,65,66,70,71,72,73], as well as their lower efficiency in the metabolic processes of OCP detoxification [68,71,74] when compared with other marine reptile vertebrates. Such substances may affect the turtles’ health status and their physiological characteristics and the process whereby hatchlings hatch in nests [75,76,77,78,79,80], and may influence the development and survival of the offspring, since the early stages of embryonic development are the most vulnerable to toxic exposure [58,66,69,81]. Throughout their life cycles, highly migratory sea turtles move between residency sites and their mating and nesting beaches; OCPS, which are associated to runoff processes from rain, can be transported to the nesting beaches from adjacent or distant areas, and eggs can absorb moisture from the environment around them, thus absorbing the toxicants dissolved in nesting beaches [33,46,82,83,84]. Additionally, given the late sexual maturity of sea turtles, nesting female turtles can incorporate OCPs into eggs during vitellogenesis and oviposition [46,71], where they can interfere with sensitive early-life development processes [28,33,58,71]. Concentrations of OCPs in eggs reflect that a developing embryo has been exposed to them at a time when their toxic effects may be especially detrimental [85]. The transportation of these contaminants in association with particulate matter represents a major pathway in the biogeochemical cycling of trace contaminants, and sea turtles have proved to be suitable bioindicators of the bioaccumulation and maternal transference of these contaminants on the ocean and also its fecundity and reproductive competence [51,73,85,86]. OCPs can accumulate over many years before being transferred from the mother to her offspring via eggs, and, after hatching, this can have serious implications for embryonic development and health [26,35,68,71,81,85,87]. In their early life stages, oviparous organisms often exhibit a greater sensitivity to chemical contaminants than in adult life stages [71,88].

The effects of anthropogenic pollution on the health and survival of marine turtles is currently one of the top twenty research topics for sea turtle conservation [17,19,68], and is considered of high importance to the recovery of turtle populations and to their conservation and management [17,18,51,53,68,88]. Many studies focusing on the concentrations of pollutants in sea turtles have examined tissues collected from dead animals, such as liver and fat; these tissues are usually used for the investigation of organic compounds because they reflect the physical and chemical properties of the target analytes [73,74,86]. Studies using plasma are very effective as a non-lethal sampling technique, and assist in efforts to monitor the long-term trends of contamination and pollutants in the fat and blood of female turtles and their respective broods, and to investigate possible maternal transference [51,89]. The maternal transference of contaminants to the eggs present in the nesting and feeding areas is not fully understood, and more investigation is needed to evaluate contaminant concentrations and their effects on hawksbill turtles’ reproductive performance. In addition, since the changes in blood chemistry can be related to their physiological state, a health assessment of nesting turtles through hematological and plasma biochemical profiles is required to obtain information on their physiological reproductive states and organ system functions [75,76,77,78,79,80]. Such information will enable the development of marine turtle management strategies in the medium and long term through informed decision making and the development of a more integrative approach to hawksbill turtle conservation. The hawksbill turtle *Eretmochelys imbricata* is a pan-tropical species listed globally as critically endangered in the International Union for Conservation of Nature Red List [20], and is legally protected by various international legislation (the Convention on International Trade in Endangered Species of Wild Flora and Fauna, the Protocol of Specially Protected Areas and Wildlife of the Wider Caribbean Region, and the Inter-American Convention for the Protection and Conservation of Sea Turtles) and national legislation in Mexico (the Endangered Species Act in the USA, NOM-SEMARNAT-059-2001) [46,77]. Hawksbill turtle conservation efforts remain primarily focused on the nesting beaches in the Yucatan Peninsula [30,31,33,38], even though nesting females spend, on average, less than 1% of their total lifetime in such habitats, recognizing that it is necessary to focus efforts towards understanding which factors (natural and anthropogenic) influence hawksbill turtles’ life stages in the marine environment. The objective of this study was to determine OCP concentrations in the blood and eggs of the hawksbill turtle *Eretmochelys imbricata*, and the relationship between the concentrations of contaminants and the nesting turtle population’s reproductive performance-specifically, maternal transference and embryonic development (total eggs, number of offspring, offspring/eggs ratio, the weight of the whole egg, weight content, and shell) in relation to sea turtle size (CCL = curved carapace length; CCW = curved carapace width). In addition, we evaluated the turtles’ health state during the breeding season and produced working reference intervals for the hematologic and plasma biochemical parameters of nesting hawksbill sea turtles along the Mexican coast (Punta Xen) [90]. Physical examinations, hematology, and the plasma biochemistry reference ranges of biochemical parameters were taken to assess and monitor the health status of sea turtles and to create suitable environmental indicators to improve the effectiveness of conservation strategies.

## 2. Material and Methods

### 2.1. Study Area

Over the last few decades, the south Gulf of Mexico and the Yucatan Peninsula have suffered from increasingly frequent disturbances from continental landmasses and river systems, urban wastewater runoff, port areas, tourism, industrial activities, pesticides from agricultural use, and other pollutants, such as metals, POPs, and hydrocarbons (from the oil industry), which contaminate water and sediments and deteriorate the environmental quality of the marine ecosystem in this region [42,43].

**Figure 1:**
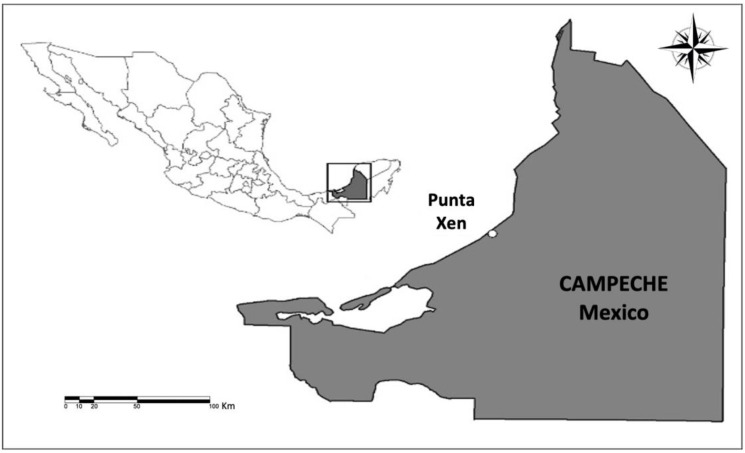
Study area. The sea turtle camp of *Grupo Ecologista Quelônios A.C*. from Punta Xen (19°12′39″ N, 90°52′09.7″ W) in Campeche Mexico.

The studded hawksbill sea turtles (*Eretmochelys imbricata*) were collected from Punta Xen Turtle camp, Campeche, a nesting area located in south-eastern Mexico on the Yucatan Peninsula, one of the most important nesting sites for the hawksbill turtle [63] (Figure 1). The samples were collected in the sea turtle camp of *Grupo Ecologista Quelonios A.C.* from Punta Xen, Campeche, Mexico, which is located on 700 hectares of natural beach (19°12′39″ N, 90°52′09.7″ W). This area hosts a few habitats, including a turtle nesting beach, a forest, mangrove habitats, and a renowned wealth of natural flora and fauna. 

### 2.2. Blood Sample Collection

During nesting seasons, whole blood and egg samples were collected from 60 nesting hawksbill turtles at Punta Xen beach in Campeche (19°12′39″ N, 90°52′09.7″ W) and were analyzed for OCPs. The license (SGPA/DGVS/03974/14) to collect the blood samples was provided by the *Secretaria de Medio Ambiente y Recursos Naturales* (SEMARNAT). The nesting female turtles’ curved carapace length and curved carapace width were measured using flexible tape [37]. Blood was collected from the dorsal cervical sinus [91] after the egg-laying process was complete. A 4 mL sample of blood was collected using a disposable syringe and immediately transferred to an EDTA Vacutainer tube (Becton Drive, Franklin Lakes, NJ, USA). For the hematological and biochemical analysis, before collecting the blood, one egg of the same hatching was collected during oviposition and wrapped in aluminum foil, transported in Ziploc bags, stored on ice, and frozen at −20 °C. For each female, a total of 5mL of blood was collected with a disposable syringe and collection tubes containing lithium heparin (Becton Drive, Franklin Lakes, NJ, USA) to prevent coagulation. The samples were centrifuged (Hermle Z206-A, Labortechnik GmbH, Wehingen, Germany) at 4000g for 10 min to obtain plasma, which was stored at −20 °C until the assay was conducted. For the hematological and biochemical analysis, the biological material that had been collected was sent to and processed in the Central Laboratory of Animal Pathology of Campeche (LACEPAC) (Campeche, México). Plasma biochemistry determinations included cholesterol, glucose, triglycerides, urea, creatinine, and uric acid [90]. All contaminant analysis was performed at the Institute of Ecology, Fishery, and Oceanography of the Gulf of Mexico (EPOMEX, Campeche, Mexico).

### 2.3. Contaminant Analysis

The organochlorine pesticides to be analyzed were selected based on the main anthropogenic activities and impacts in the area (e.g., agriculture, fisheries, waste waters), as identified in previous works [51,52,54]. OCP analysis of the blood followed the method detailed in [52]. The egg OCP analysis followed the method described by [92]. Fertile eggs were rinsed with distilled water and the contents were extracted and homogenized thoroughly. Eggs were weighed with a precision digital scale (VE-210, Velab, Mexico) (weight of the whole egg, weight content, and shell mass). The homogenized mixture was dried in an oven at 40 °C (Oven FE-291AD-Felisa, San Juan de Ocotán Zapopan, Jalisco, Mexico). Three extractions were performed in an ultrasonic bath (FS60, Fisher Scientific, Mexico). For the first extraction, 50 mL of ethyl acetate-hexane (1:1) was added, and the sample was sonicated for 1 h. The organic layer was transferred to a glass tube, and the extraction was repeated twice with 40 mL of hexane for 1 h. The samples were purified using column chromatography. The column was packed with silica gel (Aldrich Chemistry, China) (2 g), alumina (Sig-ma-Aldrich, Germany) (2 g), florisil (Sigma-Aldrich, USA) (2 g), and sodium sulfate (CTR Scientific, Mexico) (2 g). To prepare the column, 20 mL of methylene chloride (Macron Chemicals, USA), 20 mL acetone (Macron Chemicals, USA), and 20 mL hexane were added. Lastly, the sample was eluted with a 35 mL mixture of ethyl acetate: hexane (1:9). The cleaned extracts were diluted to 5 mL for analysis. The final volume of the solvent used was 0.5 mL. A mix of standards was used to analyze the OCPs (SUPELCO 47426-U CLP Organochlorine Pesticide Mix) and was divided into seven families as follows: the ΣDienes-related family (the sum of aldrin, endrin, dieldrin, endrin ketone, and endrin aldehyde), ΣHCH (the sum of α-HCH, β-HCH, χ-HCH, and δ-HCH), ΣChlordanes (the sum of cis-chlordane and trans-chlordane), ΣEndosulfans (the sum of endosulfan I, endosulfan II and endosulfan aldehyde), ΣDDTs (the sum of p,p’ DDT, p,p’ DDD and p,p’ DDE), ΣHeptachlors (the sum of heptachlor epoxide and heptachlor), and methoxychlor. The limit of detection for each family of compounds was in μg g^−1^ (HCHs—0.007; Aldrin—0.0018; DDTs—0.01; Chlordanes—0.009; Endosulfans—0.007; Heptachlors—0.013; Methoxychlor—0.01) [11].

### 2.4. Instrumental Analysis

A Varian 3800 gas chromatograph was used to quantify the contaminants equipped with a Ni63 electron capture detector and a DB-5 (5% phenyl) methylpolysiloxane column measuring 30 m × 0.25 mm × 0.32 mm. The injector temperature was 270 °C and the detector was 300 °C. The initial temperature of the oven was 60 °C and it increased at a rate of 2 °C/min until reaching 300 °C, and this temperature was maintained for 5 min. The flow of nitrogen into the column was 2 mL/min and a makeup of 30 mL/min. Quantitative data were obtained by calculating the area under the curve with the Star Chromatography Workstation software (version 6) and using the calibration standards. Laboratory blanks were analyzed for quality assurance. Chicken egg samples were used in triplicate. One milliliter of a 200 ng/mL pesticide mix (SUPELCO) was added to the samples before the extraction, and they were subsequently refrigerated for 48 h. One of the subsamples was not spiked with the standard as a positive blank. Afterward, the contaminants were extracted and processed in a process identical to that used for the rest of the samples, with a recovery of >85%. 

### 2.5. Statistical Analysis

For the hematological and biochemical statistical analysis (mean, standard deviation, minimum, and maximum), R version 3.2.3 was used (R Core Team (2015)). The statistical significance level was set at *p* < 0.05. The distribution of all parameters was tested for normality using a Shapiro-Wilk test. Correlations between curved carapace length data and the biochemical parameter values of this study were evaluated using Pearson’s chi-squared test. A non-parametric statistic was applied to data: the differences between years were accessed using the Mann-Whitney U Test and the differences between tissues were evaluated by applying the Wilcoxon signed rank test [93]. A Spearman correlation was performed to analyze the degree of association between the OCP concentrations and the morphometric parameters, the number of offspring, and offspring/egg ratios. The Spearman correlation was performed for *p* < 0.05. Non-metric multidimensional scaling (MDS) was used to produce two-dimensional ordination plots. The Bray-Curtis coefficient was used to construct the similarity matrix from the square-root-transformed data. For the tests, 9999 permutations were used. A significance level (*p*) of <0.05 was considered. All preliminary data analyses and non-parametric tests were performed using SPSS (IBM version 21). The MDS tests were performed using PRIMER with PERMANOVA+ software (PRIMER v6 and PERMANOVA+ v1, PRI-MER-E Ltd.).

## 3. Results 

### 3.1. Concentrations and Patterns

Sixty hawksbill sea turtles were sampled with a mean CCL of 89.87 ± 6.36 cm (75.50–101.00 cm). A gross clinical examination did not detect obvious abnormalities, such as tumors or injuries, in any of the sampled turtles. The size and egg contents in hawksbill turtles showed a significant increase in shell mass between 2014 and 2015 (Table 1).

### 3.2. Hematology and Plasma Biochemistry 

**Figure 2:**
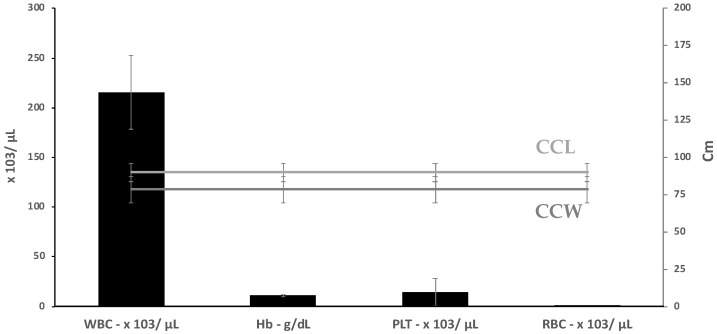
Hematological values (mean ± SD) and ranges in foraging hawksbill turtles nesting in Punta Xen, Yucatan Peninsula, Mexico.

**Figure 3:**
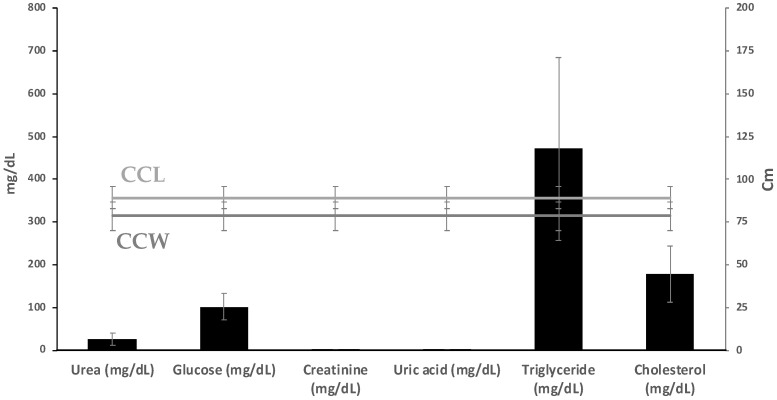
Plasma chemical values (mean ± SD) and ranges in foraging hawksbill turtles nesting in Punta Xen, Yucatan Peninsula, Mexico.

The range, mean, and standard deviation (Mean ± SD) of biometric data and biochemical parameters of female foraging and nesting turtles are given in Figure 2, and the results of the hematologic tests are provided in Figure 3. The mean PCV was 0.80, with a range of 0.20 ± 2.50, and the mean WBC count was 215.50, with a range of 101.20 ± 250.70. 

Plasma biochemistry data are provided in Figure 3, with values reported for blood collected in lithium heparin. Most of the biochemical parameters have a significant correlation with biometric factors (*p* < 0.05) with CCL: urea (*p*-value = 0.494); creatinine (*p*-value = 0.4227), glucose (*p*-value = 0.4554), cholesterol (*p*-value = 0.08054), uric acid (*p*-value = 0.9309) and triglyceride (*p*-value = 0.4908) [90].

### 3.3. Relationship between Egg and Blood Concentrations

In blood, ΣDienes and ΣDDTs were the predominant OCPs found in 2014, and ΣHCHs and ΣDDTs predominated in 2015 (Table 2). ΣDienes were detected in all blood samples in 2014. ΣDDTs were the second most frequent OCP class measured each year, represented mainly by the metabolites p,p’ DDT, p,p’ DDD, and p,p’ DDE, which occurred in 91.6% of the samples. A significant difference was observed between the years for the concentrations of ΣDienes, ΣDDTs, and Methoxychlor in blood. No significant differences were observed in the blood concentrations between the years for the other contaminant classes. In eggs, ΣHCHs and ΣDienes were the most frequent OCP classes, found in 98.3% and 93.3% of samples, respectively, in 2014. Meanwhile, ΣHCHs (88.3%) were predominant in 2015. ΣChlordanes were the third most frequent OCPs class measured, represented mainly by the metabolites cis-chlordane and trans-chlordane; they occurred in 88.3% of samples in 2014. There were significant differences in concentrations of ΣDienes, ΣChlordanes, and ΣDDTs in eggs between 2014 and 2015. No significant differences were observed between the years for the remaining OCPs.

Considering the eggs/blood ratios, significant differences between years were found for ΣHCHs, ΣDienes, ΣChlordanes, and ΣDDTs (Table 3). Significant differences between tissues (eggs and blood) were observed for ΣHCHs and ΣDienes. Pearson correlations are presented in Table 4. Significant correlations were observed between ΣDDTs and both the weight of the whole egg (g) and the total weight content (g). The results also suggest that the shell (g) is positively correlated with the levels of ΣHCHs, ΣDienes, ΣChlordanes, ΣEndosulfans, and Methoxychlor measured in eggs.

The observed Pearson correlations between the number of offspring and OCPs measured in the blood (Table 5) suggest that some of the detected contaminants, including ΣDienes, ΣEndosulfans, and Methoxychlor, may have significant negative effects on the turtles’ reproductive success. The levels of ΣHCHs found in eggs seem to have significant negative effects on hatching success. No significant correlations were observed for the remaining OCPs measured in eggs.

**Figure 4:**
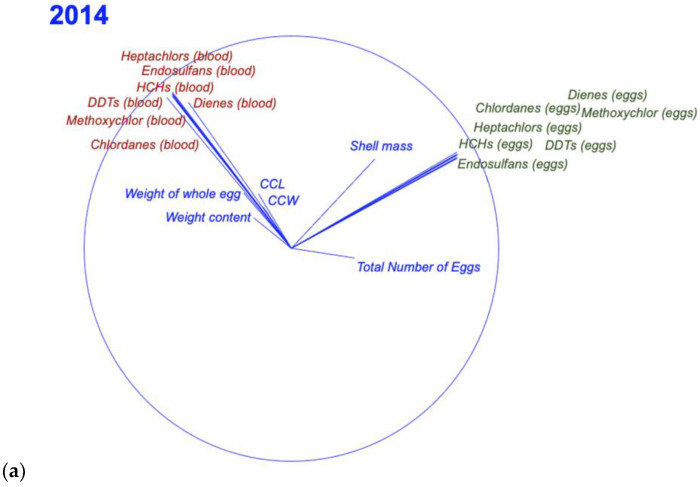

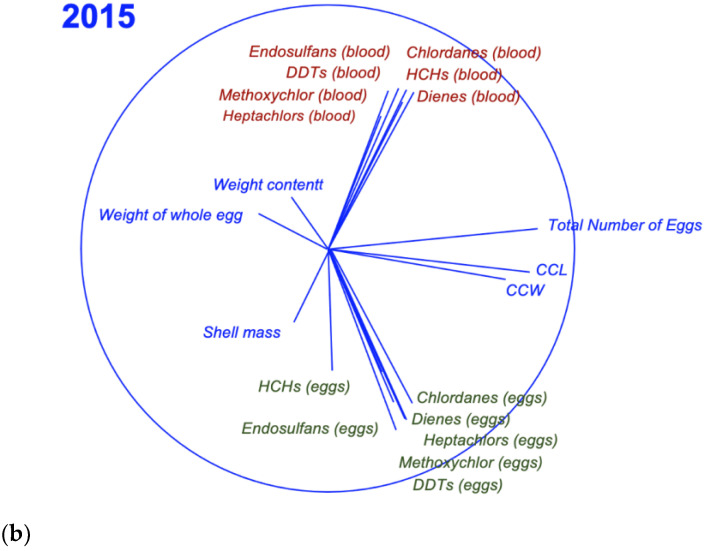
MDS output representing the correlations between the number of eggs, the morphometric variables (blue), and OCPs detected in the blood (red) and eggs (green), measured in (**a**) 2014; 2D stress: 0.07 and in (**b**) 2015; 2D stress: 0.15.

An MDS was used to produce two-dimensional ordination plots for two years (2014 and 2015). The results for the two consecutive years indicated a clear separation between the three major blocks (eggs, blood, and morphometrics) (Figure 2a,b). Both MDS analyses suggest a clear separation between the three major groups, including OCPs in eggs, OCPs in blood, and morphometric parameters, particularly in the 2014 data (Figure 4a,b), thus reinforcing the correlations presented in Table 4. In both years, the eggshell weight was positively correlated with the OCPs measured in the eggs.

## 4. Discussion

The present study focused on the effects of environmental pollution, and in particular on OCP contamination, in order to assess the potential risk that they pose to turtles’ health; our findings are significant for management strategies and conservation. Our study provides new eco-toxicological data for OCs in live hawksbill turtles nesting at Punta Xen turtle camp (Yucatan, Mexico), and constitutes one of the few studies that has tracked the hawksbill turtles of the Yucatan Peninsula, which are part of a broadly distributed group and one of the largest in the Atlantic Basin [94]. Due to inadequate conservation measures on the nesting beaches [18,33,34], and due to their unsustainable exploitation for food and tortoiseshell, this species has historically suffered population declines [38,94].

This research provides additional baseline data on contaminant concentrations in sea turtle blood and eggs, and offers evidence of possible maternal transference. The observed results show significant differences between maternal blood and egg OCPs concentrations (ΣHCHs and ΣDienes), indicating that these chemicals are transferred from nesting females to eggs and, ultimately, to hatchlings. In 2014, the ΣHCHs in blood and eggs and the ΣDDTs in blood were similar to the levels observed in [85] in leatherback turtles, *Dermochelys coriacea*, nesting in French Guiana. The values of ΣDDTs detected were higher in the eggs of loggerhead turtles [72] than in leatherback turtles [89] and the hawksbill turtles from this study. However, the concentrations of ΣDDTs in the blood of hawksbill turtles were higher than the concentrations in leatherback turtles [85,89]. The relationship found between OCP concentrations in eggs and blood was positive for ΣHCHs, ΣDienes, and ΣChlordane, following the results obtained in [85], which showed positive correlations between concentrations of ΣDDT and p,p’ DDE (*p* = 0.0009 and *p* = 0.0001, respectively) in the leatherback turtles’ blood and eggs. Another study [88] found that total PCBs, 4,4-DDE, total PBDEs, and total chlordane were significantly and positively correlated between blood and eggs, suggesting that lower levels of lipophilic compounds appear to more readily transfer from females to their eggs. Significant correlations between maternal blood and eggs were found for PCBs, PBDEs, HCH, trans-chlordane, and mirex [85]. Similar correlations were observed between eggs and hatchlings’ blood [94]. A relationship between hawksbill turtles and the concentrations of OCPs was previously reported [51]. However, in the present investigation, no significant relationships (*p* < 0.05) were found between the CCL and CCW and the concentration of OCPs measured in hawksbill turtles. [51] Another study observed a negative correlation between the size of the turtles and the concentration of OCPs in the eggs and blood of the hawksbills. The hawksbill turtles have a complex life cycle [95]; thus, these differences can be explained by the turtles’ life history and/or seasonal and age-related diet shifts (shifting from omnivore to herbivore) [96], leading to a dilution of the concentration of OCPs (and other contaminants) as the animals grow. Another possible explanation is related to age; considering that size and age are related, a possible maternal transfer could also be a factor for the elimination or partial elimination of OPCs [51].

These results are also in good agreement with the MDS for both years, with three major groups (eggs, blood, and morphometric parameters) being, in general, unrelated to each other. However, the MDS results also indicated that the eggshell weight is positively correlated with the OCPs measured in eggs. The variation in the OCP profiles observed in the present study indicates that animals are being exposed to different types and concentrations of OCPs. Nevertheless, care is needed when comparing values with other studies, because concentrations of OCPs in sea turtles are driven by complex interactions between biological (e.g., age class, sex, body condition, season (nesting or breading)), anthropogenic (e.g., sources of contamination and other pressures) and environmental (e.g., temperature, salinity, precipitation) factors [51,67,97]. Another recurring issue arises from the comparison of different OCPs using distinct laboratory methods and concentration units [98], resulting in biased interpretations.

The MDS results also support this important aspect of our research. Significant correlations between maternal blood, eggs, and hatching success and OCP concentrations indicate that these chemicals are being transferred from nesting females to their eggs and have negative effects on hatchlings [94]. Due to the lipophilic properties of OCPs, these chemicals are likely to be transferred from nesting females as lipids, which are mobilized for yolk production [94,99]. In recent years, several studies have reported the potential effects that OCPs may have on the reproduction and health of wild animals, compromising the future of some of these species [68,69]. Moreover, [85] and [89] found that both PCBs and PBDEs are maternally transferred to eggs and hatchlings in leatherback turtles, as we found in this study. The authors of [70] provided the first evidence that POPs can affect health parameters and may have sub-lethal effects in the loggerhead sea turtle; there is also evidence that certain levels of POPs can have negative effects on the reproductive success of green turtles [94]. A strong negative correlation between the sum of PBDE concentrations and the hatching success rate was reported by [67]. A detrimental effect on the turtles’ hatching success rate can result in demographic structure shifts, with a severe impact on population persistence and survival.

The general health status of the turtles in this study, as measured by urea, serum, creatinine, glucose, uric acid, and cholesterol, and triglyceride, was rated within normal ranges and was similar to other species’ normal values in other latitudes, which could indicate that the turtles have good energy levels and body conditions suitable for nest-building activity, with all of these turtles able to successfully come ashore to nest [9,66,75,76,77,78,79,80]. However, the obtained results also indicate that OCPs affect the health of organisms, the nesting and reproductive performance of hawksbill turtles, their fertility, and the development of the population of eggs and reproductive performance, specifically in terms of maternal transference and embryonic development. In the present investigation, significant negative correlations were found between ΣDienes measured in the blood samples and the number of offspring. Furthermore, the levels of ΣEndosulfans and Methoxychlor measured in the blood and the levels of ΣHCHs found in eggs seem to have significant negative effects on the hatching success. Moreover, the eggshell weight was found to be positively correlated with the majority of OCPs (except for ΣDDTs and ΣHeptachlors) measured in eggs. These results clearly indicate that some of the OCPs detected in mothers’ blood and eggs have negative effects on the reproductive success of hawksbill turtles, as has been found in other studies [67,72]. Moreover, they indicate a significant increase in consecutive years and did not differentiate between tissues, which also suggests that the transfer of OCPs to hatchlings may occur beyond the compound-specific contaminant, reducing survival through several mechanisms including acute mortality. Considering the results of other studies, these elevated levels of contaminants likely contribute to the deaths of young organisms and mortality in the initial stages of development [100,101,102]. The transference of accumulated contaminants to the eggs during the life of an adult female turtle may result in lethal levels being transferred to the developing embryo, especially in the first clutch of eggs, which can impair the reproductive rate by many mechanisms, most significant of which is the disruption of the endocrine system. This disruption by contaminants can result in abnormal development, altered sex ratios, and a reduction in reproductive rates, as observed in other animals [103,104]. In the early life stages of oviparous vertebrates, such as turtles, the developing embryo is likely most sensitive and susceptible to anthropogenic contaminants, compared with adults [105,106]. According to [107], exposure to POPs during the early life stages of several oviparous organisms is similar to the exposure of the adults (who deposit the eggs). This means that, if animals are more susceptible to these contaminants during the earlier stages of life, the toxic effects are more likely to occur in developing embryos than in the adult organisms.

These results represent important contributions to the development of conservation strategies for sea turtles on the Mexican coast, considering the regional context in addition to the global situation [108]. Findings such as these are important because they allow for the assessment of risks and threats to sea turtles, and assist important projects such as the development of conservation strategies and structures to define conservation priorities and develop ecological information, as well as assisting in decision-making processes, as in the case of legal and social causes to balance technical, governance, and social factors [109]. Conservation strategies need to focus on regional assessments and on differentiating each species by addressing stream-specific reasons for their decline. Thus, long-term, accurate, and comparable monitoring data are needed. This implies the standardization of monitoring protocols, which is essential for efforts directed toward understanding which (natural and anthropogenic) factors influence the life stages of hawksbill turtles in the marine environment. This information strengthens the capacity of sea turtle management strategies in the medium and long term, and will inform decision making and allow for the development of a more integrative approach to hawksbill conservation (protection and management) and long-term conservation measures.

Even with the conservation programs for sea turtles in this region (the WWF, Marine Turtle Action Plan, Latin America and the Caribbean: 2015–2020) [110], it is recognized that natural science research alone is insufficient to find solutions to complex conservation problems that have social dimensions [111]. Hawksbills are threatened by the direct legal and illegal capture of meat and eggs, and the international trade of their shells, which are used for decorative purposes around the world [112]. Due to coastal development, sand erosion, artificial lights, and pollution, hawksbill nesting and foraging habitats have been lost or modified in Latin America and the Caribbean. Climate change is likely to further alter conditions at existing nesting and foraging sites [110]. It is still necessary to strengthen these programs and raise awareness in the communities around the main beaches to involve them in the work of protection and conservation. Due to their highly migratory and geographically widespread nature, sea turtles require transboundary conservation strategies that often include multiple institutions and government bodies, spanning multiple geopolitical boundaries, agreements, and instruments at local, national, and international scales [59,60,61]. Conservation strategies must include ongoing research, the management of local turtle populations, and the education of local people, including encouraging fishermen to release turtles that are accidentally caught. Data, such as those related to concentrations of contaminants, are also relevant; they are necessary for investigations of the geographic trends related to these concentrations and the potential health effects caused by these contaminants. Such investigations would include the relationships of these compounds with hatching success, embryo abnormality rates, hatch survival rates, sex ratios, and hatch growth rates in sea turtle development. However, it is important to keep in mind that conservation priorities vary widely depending on the goals and values of different governing bodies, NGOs, researchers, funding bodies, and other stakeholders [17,18,19]. Considering marine turtles’ biological features and human-induced threats, conservation actions also need to be sustained over decades, conducted over vast areas, be relevant to diverse marine and terrestrial environments, and involve international cooperation and coordination. Activities and studies must include research, strengthening environmental education, local management strategies, interaction with local fishermen, encouraging the safe release of turtles accidentally caught in fishing nets, discussion programs for the conservation of wildlife threatened by extinction, and voluntary training courses [105]. Programs must also emphasize information exchange between science, policy, and public participation in the design and implementation of conservation actions.

## 5. Conclusions

This study provides a basis for the monitoring of nesting sea turtles’ general health status and blood and eggs for contaminant concentrations, and the toxic effects of these contaminants in hawksbill turtles. Our results also suggest that OCPs can be maternally transferred in hawksbill turtles, and provide an important baseline of OCP concentrations for nesting and stranded hawksbills in south-eastern Mexico. However, further research is needed to confirm this hypothesis. Furthermore, dedicated studies must be undertaken to examine the source of these contaminants and, more importantly, to determine the population-level effects of these compounds on this endangered species. Future research is required to investigate geographical trends in contaminant concentration levels at broader temporal and spatial scales; this work must prioritize regional assessments, as well as the potential health effects of these contaminants on sea turtles’ development. The present work contributes to ongoing effort to understand and mitigate these threats, facilitating the development of appropriate management and conservation tools for wild hawksbill turtles.

## Figures and Tables

**Table 1:** Summary of the parameters measured in hawksbill sea turtles nesting at Punta Xen: size (cm) and egg contents (g) of hawksbill turtles (mean ± standard error of the mean; min; max). * Asymptotic *p*-value; *p* < 0.05.

Parameters	First Year Nesting Season			Second Year Nesting Season			Mann-Whitney U Test	
	Mean ± SEM	Min	Max	Mean ± SEM	Min	Max	U	*p* *
**CCL (cm)**	89.953 ± 1.155	76	101	89.233 ± 1.205	75.5	100.0	407	0.671
**CCW (cm)**	77.881 ± 1.652	39	90	79.550 ± 1.142	70.0	93.5	393.5	0.577
**Total Eggs**	133.56 ± 3.882	83	178	136.14 ± 5.391	87.0	194.0	416	0.765
**Weight of Whole Egg (g)**	30.632 ± 0.479	25	36.2	30.680 ± 0.483	25.8	37.8	420.5	0.826
**Weight Content (g)**	28.413 ± 0.463	23.1	33.2	27.917 ± 0.462	23.3	35.1	387.5	0.471
**Shell Mass (g)**	2.382 ± 0.068	1.8	3.3	2.763 ± 0.108	2.10	4.60	261	0.008

CCL = curved carapace length; CCW = curved carapace width; SEM = standard error of mean.

**Table 2:** Summary of the parameters measured in hawksbill sea turtles nesting at Punta Xen: organochlorine contaminant concentrations in eggs and blood (ηg/g^−1^). * Asymptotic *p*-value; *p* < 0.05; statistically different values are indicated in bold.

		Blood ^a,1^		Mann–Whitney U Test		Eggs ^a^		Mann–Whitney U Test	
OCPs	N	First Year Nesting Season	N Second Year Nesting Season	U	*p* *	N First Year Nesting Season	N Second Year Nesting Season	U	*p* *
**ΣHCHs**	29	0.204 ± 0.062	30 1.948 ± 0.930	314	0.060	29 0.521 ± 0.066	30 3.429 ± 1.711	319.5	0.071
**ΣDienes**	29	0.779 ± 0.091	30 0.882 ± 0.449	273.5	**0.009**	29 0.342 ± 0.089	30 2.567 ± 1.522	302.5	**0.045**
**ΣChlordanes**	29	0.129 ± 0.058	30 0.468 ± 0.211	401	0.577	29 0.221 ± 0.056	30 1.488 ± 0.840	308	**0.048**
**ΣDDTs**	29	0.290 ± 0.095	30 1.593 ± 0.631	311	**0.047**	29 0.203 ± 0.066	30 2.072 ± 1.177	299.5	**0.034**
**ΣHeptachlors**	29	0.057 ± 0.016	30 0.767 ± 0.417	418	0.792	29 0.110 ± 0.029	30 1.100 ± 0.603	365	0.258
**ΣEndosulfans**	29	0.149 ± 0.051	30 1.166 ± 0.545	408	0.680	29 0.208 ± 0.066	30 1.681 ± 0.999	334.5	0.111
**Methoxychlor**	29	0.080 ± 0.023	30 0.566 ± 0.244	307.5	**0.041**	29 0.079 ± 0.027	30 0.677 ± 0.425	366	0.228
**TOTAL**		1.690 ± 0.373	7.395 ± 3.378			1.688 ± 0.361	13.016 ± 7.229		

N = sample size; ^a^ = mean ± SEM; ^1^—blood values were made available by [52].

**Table 3:** Relationship between organochlorine contaminant concentrations in eggs (ng/g wet mass) compared with concentrations in blood between the years under consideration. (a) Mann–Whitney U test (* asymptotic *p*-value; <0.05); (b) Wilcoxon signed rank test (** asymptotic *p*-value; *p* < 0.05); statistically different values are indicated in bold.

	Eggs/Blood Ratio		Between Years ^(a)^		Between Tissues ^(b)^	
OCPs	First Year Nesting Season ^a^	Second Year Nesting Season ^a^	F	*p* *	F	*p* **
**ΣHCHs**	5.838 ± 1.877	41.132 ± 36.380	254.5	**0.007**	−2.811	**0.005**
**ΣDienes**	0.571 ± 0.170	47.587 ± 29.849	212.5	**0.000**	−2.381	**0.017**
**ΣChlordanes**	1.302 ± 0.603	12.458 ± 10.115	301.5	**0.045**	−1.721	0.085
**ΣDDTs**	1.817 ± 0.598	9.736 ± 6.717	310	**0.049**	−0.654	0.513
**ΣHeptachlors**	2.328 ± 0.829	3.668 ± 1.874	334	0.069	−0.872	0.383
**ΣEndosulfans**	2.843 ± 1.153	2.851 ± 2.246	354	0.157	−1.038	0.299
**Methoxychlor**	1.591 ± 0.667	3.694 ± 2.684	349.5	0.064	−0.698	0.485
**TOTAL**	2.050 ± 0.5286	33.8608 ± 21.8481				

^a^ = mean ± SEM.

**Table 4:** Pearson correlations between the morphometric parameters and OCPs measured in eggs.

OCPs	CCL (cm)	CCW (cm)	Total Eggs	Weight of Whole Egg (g)	Weight Content (g)	Shell (g)
**ΣHCHs**	0.022	0.036	0.119	0.159	0.110	0.270 *
**ΣDienes**	−0.060	−0.028	0.001	−0.099	−0.114	0.271 *
**ΣChlordanes**	−0.130	−0.106	0.017	−0.030	−0.062	0.279 *
**ΣDDTs**	−0.026	−0.226	0.096	−0.270 *	−0.267 *	0.188
**ΣHeptachlors**	−0.114	−0.098	0.040	−0.006	−0.026	0.205
**ΣEndosulfans**	−0.114	−0.096	0.043	−0.220	−0.225	0.265 *
**Methoxychlor**	−0.149	−0.147	0.017	−0.150	−0.163	0.297 *

*. Correlation is significant at the 0.05 level.

**Table 5:** Pearson correlations between the number of offspring and OCPs measured in blood and eggs. The same correlations between the hatching success and the contaminants analysed in both blood and eggs are also presented.

		Number of Offspring	Hatching Success
OCPs in Blood	ΣHCHs	0.051	0.011
	ΣDienes	−0.369 *	−0.349
	ΣChlordanes	0.299	−0.130
	ΣDDTs	0.283	−0.315
	ΣHeptachlors	0.269	−0.234
	ΣEndosulfans	0.234	−0.426 *
	Methoxychlor	0.293	−0.350 *
OCPs in Eggs	ΣHCHs	−0.218	−0.364 *
	ΣDienes	0.031	0.112
	ΣChlordanes	0.236	0.271
	ΣDDTs	0.175	0.028
	ΣHeptachlors	0.121	0.070
	ΣEndosulfans	0.144	0.013
	Methoxychlor	0.042	0.120

* Correlation is significant at the 0.05 level.

## Data Availability

Not applicable.
